# Potential Mechanisms Linking Excessive Testosterone to PMOS: Insights from Network Toxicology and Machine Learning

**DOI:** 10.3390/metabo16090663

**Published:** 2026-09-09

**Authors:** Chao Li, Zhe Su, Yiqian Li, Huili Liu, Mengyi Zheng, Hanjing Zhou, Cheng Wei, Feng Zhou, Cuiyu Yang, Chen Tang, Bin Chen

**Affiliations:** 1 Assisted Reproduction Unit, Department of Obstetrics and Gynecology, Sir Run Run Shaw Hospital, School of Medicine, Zhejiang University, Hangzhou 310016, China; 2 Zhejiang Key Laboratory of Precise Protection and Promotion of Fertility, Hangzhou 310016, China; 3 Zhejiang Provincial Clinical Research Center for Reproductive Health and Disease, Hangzhou 310016, China

**Keywords:** polyendocrine metabolic ovarian syndrome, testosterone, network toxicology, machine learning, molecular docking

## Abstract

**Background/Objectives:** Polyendocrine metabolic ovarian syndrome (PMOS) is characterized by hyperandrogenism, particularly excessive testosterone, as a core clinical feature and a key pathogenic metabolite, yet its molecular mechanisms remain incompletely understood. **Methods:** This study integrated multi-omics data from Gene Expression Omnibus (GEO) databases with network toxicology, weighted gene co-expression network analysis (WGCNA), and machine learning to identify testosterone-associated core genes in PMOS. **Results:** Differential expression analysis and WGCNA yielded 42 candidate genes, from which five core genes, including GK5, CYP3A5, EGLN3, VCAM1, and AGTR1, were prioritized as top predictive features through ensemble modeling (RF + XGBoost). Molecular docking predicted favorable testosterone binding conformations. Regulatory network and drug enrichment analysis additionally predicted several upstream transcription factors, hub miRNAs, and potential repurposable drugs. **Conclusions:** These findings proposed a computational framework for a multi-target molecular landscape linking testosterone to PMOS. The identified genes, regulatory networks, and candidate drugs provided prioritized hypotheses for mechanistic exploration and future evaluation of potential diagnostic and therapeutic applications in hyperandrogenism-related PMOS.

## 1. Introduction

Polyendocrine metabolic ovarian syndrome (PMOS), previously known as polycystic ovary syndrome (PCOS), was chosen as the accurate new name based on international consensus, to reflect the disorder’s diverse metabolic and endocrine features [1]. PMOS is a common and heterogeneous endocrine disorder affecting women of reproductive age [2]. According to the 2023 International Evidence-based Guideline, PMOS presents with hyperandrogenism, ovulatory dysfunction, and polycystic ovarian morphology or elevated anti-Müllerian hormone levels [2,3]. PMOS affects approximately 10–13% of women worldwide under the Rotterdam criteria and imposes substantial reproductive, metabolic, psychological, long-term health, and economic burdens [2,4]. Clinically, PMOS comprises distinct phenotypes defined by different combinations of diagnostic features, and these phenotypes differ in endocrine characteristics, metabolic risk, clinical severity, and possible etiopathogenesis [2,3,5,6,7]. Therefore, the pathogenic mechanisms underlying different PMOS phenotypes should be investigated separately to support phenotype-specific mechanistic interpretation and intervention development.

Among women with PMOS, 40–80% of patients present with hyperandrogenism, based on epidemiological data [8]. Hyperandrogenemia is widely considered an important feature and potential contributor to PMOS pathophysiology, and genetic analyses from multiple studies have shown a complex relationship between testosterone levels and health as well as disease [9]. Specifically, elevated testosterone levels have a more pronounced impact on reproductive and metabolic health in women [10]. In PMOS, high androgens impair follicular maturation by promoting primary follicle recruitment, which increases the number of gonadotropin-independent small antral follicles, thereby blocking dominant follicle growth and ovulation [5,7]. In addition, hyperandrogenemia has been associated with metabolic disturbances in PMOS by affecting peripheral metabolic tissues—including adipose tissue, liver, pancreas, muscle, and brain—contributing to a wide range of complications, from obesity to insulin resistance [11]. Yet, how hyperandrogenism leads to dysfunction in PMOS is still poorly understood, and the underlying mechanisms remain largely unclear.

Existing evidence indicates that functional variants and polymorphisms in CYP11A1, CYP17, CYP19, STAR, HSD17B5, INSL3, AR, and SHBG disturb testosterone homeostasis and trigger hyperandrogenemia, acting as key genetic contributors to the development of PMOS [12]. These findings suggest that disrupted steroid biosynthesis, androgen metabolism, hormone transport, adipose androgen generation, and androgen receptor signaling may contribute to the pathogenesis of hyperandrogenic PMOS [12,13,14]. However, existing evidence is still limited by inconsistent genetic associations, ethnic heterogeneity, and a predominant focus on individual genes or linear pathways [12]. The rise of network toxicology has reshaped our approach to deciphering complex interactions between compound exposure and outcome. Network toxicology combines bioinformatics, big data, and genomics to explore toxicological pathways and disease mechanisms [15]. It uses systematic network analysis to reveal how chemicals interact with biological targets and cause adverse reactions, shifting the paradigm toward a “multi-target, single drug” model [15]. Molecular docking predicts how small molecule ligands bind to active sites on biological macromolecules based on their spatial conformation. This computational method helps evaluate therapeutic or toxic effects and is commonly used to predict interactions between compound toxins and biomolecules [15,16]. Therefore, integrating testosterone-related target databases with PMOS-associated transcriptomic databases may help identify shared molecular nodes and pathogenic pathways specific to hyperandrogenism-related PMOS.

**Figure 1:**
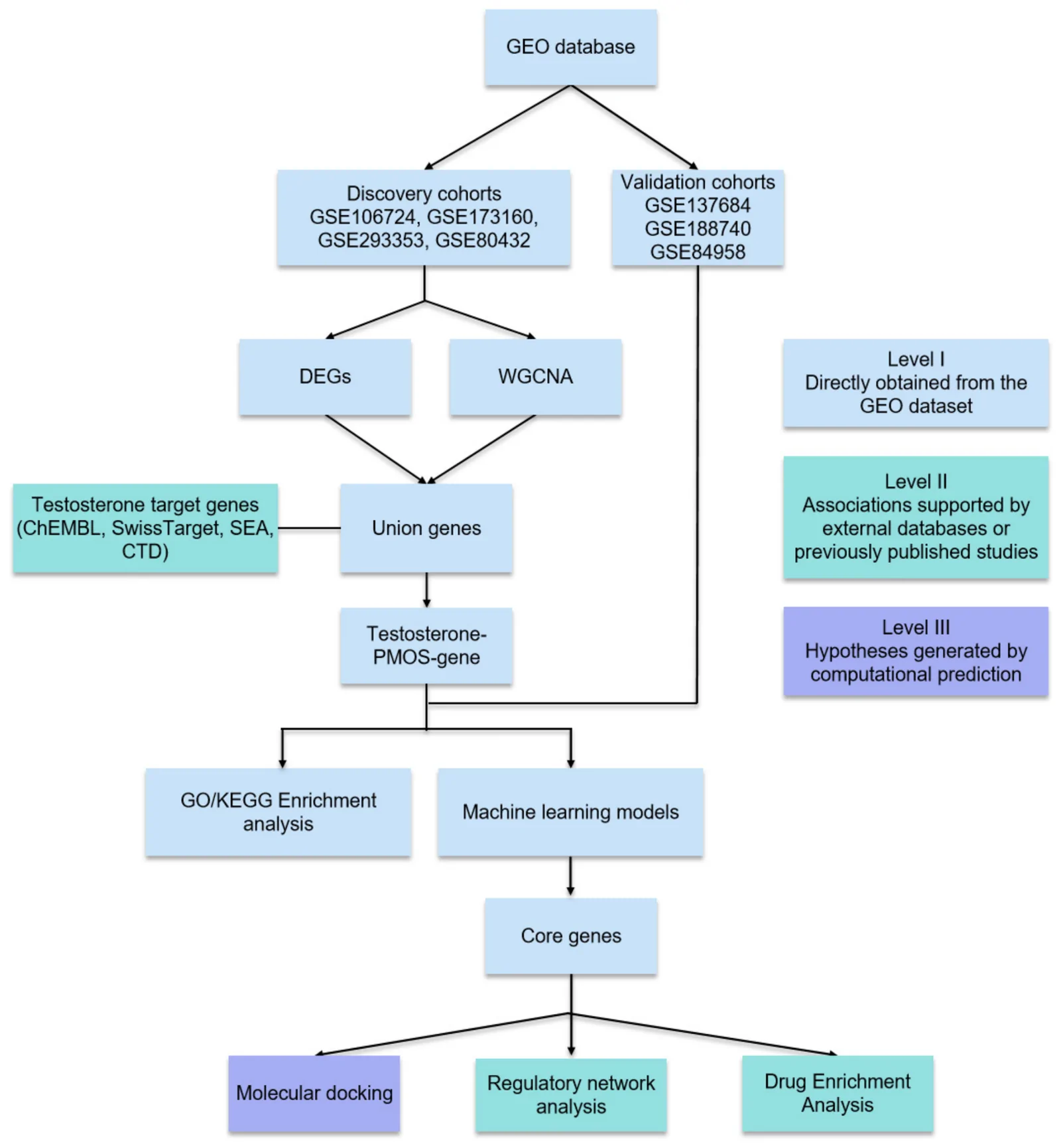
Flowchart of the study. Colors in this flowchart denote three distinct levels of evidence generated in this study: Blue (Level I): Directly obtained from the GEO dataset. Green (Level II): Associations supported by external databases or previously published studies. Purple (Level III): Hypotheses generated by computational prediction. The arrows indicate the logical sequence of the analytical pipeline.

In this study, we applied an integrated network toxicology strategy to investigate the molecular mechanisms of testosterone-associated PMOS. By constructing testosterone-associated PMOS networks from multi-omics data, prioritizing hub molecules via machine learning and enrichment analyses, and validating target binding via molecular docking, we sought to map testosterone’s disease network and prioritize candidate molecular targets that may inform chemoprevention and individualized treatment for populations at high risk of PMOS. The workflow of the analysis is shown in Figure 1.

## 2. Materials and Methods

### 2.1. Collection of the Chemical Constituents and Associated Targets of Testosterone

The characterization of testosterone was accomplished by integrating data from multiple databases. Physicochemical properties and biological parameters were systematically compiled from PubMed, whereas conventional two-dimensional structural descriptors (SMILES: C[C@]12CC[C@H]3[C@H]([C@@H]1CC[C@@H]2O)CCC4=CC(=O)CC[C@]34C) were obtained from the PubChem database. A quadruple strategy was subsequently applied for target prediction—ChEMBL database (https://www.ebi.ac.uk/chembl, accessed on 26 May 2026): Interaction profiling between ligands and receptors; SwissTargetPrediction (http://www.swisstargetprediction.ch, accessed on 26 May 2026): Prediction leveraging chemical genomics principles; Similarity Ensemble Approach (SEA) (https://sea.bkslab.org, accessed on 26 May 2026): Ligand-based target similarity searching; and the Comparative Toxicogenomics Database (CTD) (https://ctdbase.org, accessed on 26 May 2026): Chemical gene–disease interaction mapping. Only targets within the human (*Homo sapiens*) proteome were considered for prediction. The target details of testosterone are shown in Supplementary Tables S1–S4.

### 2.2. Differential Expression Analysis and Batch Effect Correction

Multiple Gene Expression Omnibus (GEO) transcriptomic datasets (GSE106724, GSE173160, GSE293353, and GSE80432) were downloaded from the GEO database (https://www.ncbi.nlm.nih.gov/geo, accessed on 15 April 2026) and integrated [17]. Table 1 presents the relevant information for each dataset. Datasets were initially processed for background correction, quantile normalization, and log_2_ transformation. To remove technical heterogeneity arising from different study origins, batch effects were corrected using the removeBatchEffect function from the limma package (version 3.62.2) in R software (version 4.4.3) [18,19]. Correction efficacy was evaluated by boxplots of all samples and principal component analysis (PCA) before and after adjustment. Following batch removal, differential expression analysis between PMOS and control samples was conducted. Genes with |log_2_ fold change| > 0.585 (corresponding to a 1.5-fold change) and Benjamini–Hochberg adjusted *p*-value < 0.05 were considered significantly differentially expressed [20]. Volcano plots were generated using the ggplot2 package (version 4.0.3) [21]. For heatmap visualization, the top 30 upregulated and top 30 downregulated genes (ranked by adjusted *p*-value) were selected.

### 2.3. Weighted Gene Co-Expression Network Analysis (WGCNA) and Candidate Gene Identification

Weighted gene co-expression network analysis (WGCNA) was performed on the batch-corrected expression matrix using the WGCNA package (version 1.74) [22,23]. A soft-thresholding power was selected to ensure scale-free topology. Co-expression modules were identified by hierarchical clustering based on topological overlap matrix (TOM) dissimilarity, followed by dynamic tree cutting and merging of similar modules. Module eigengenes were correlated with clinical traits to identify disease-relevant modules. For the selected module, module membership (MM) and gene significance (GS) for PMOS were calculated. Finally, the union between differentially expressed genes (DEGs) and genes from the PMOS-associated module was obtained via Venn analysis with the ggvenn package (version 0.1.19) [24].

### 2.4. Identification of Testosterone-Associated PMOS Genes and Functional Enrichment

The overlapping genes between testosterone-associated genes and PMOS-associated genes were identified using Venn analysis with the ggvenn package (version 0.1.19) as well [24]. Subsequently, the testosterone–PMOS–gene interaction network was constructed and visualized using Cytoscape software (version 3.10.4) [25]. For functional annotation, Gene Ontology (GO) enrichment analysis (biological process, cellular component, and molecular function) and Kyoto Encyclopedia of Genes and Genomes (KEGG) pathway enrichment analysis were performed on the overlapping genes using the clusterProfiler package (version 4.14.6) with the org.Hs.eg.db package (version 3.21.0) as the annotation database [26,27]. Statistical significance was set at *p* < 0.05. Results were visualized as bar plots (GO) and bubble plots (KEGG), with gene ratio and *p*-value indicated by point position and color gradient.

### 2.5. Machine Learning-Based Core Genes Screening

We built a multi-algorithm prediction framework to screen for testosterone-associated potential diagnostic markers in PMOS. Using expression profiles from the training set, we ran dozens of classical machine learning algorithms: Lasso, Support Vector Machine (SVM), Random Forest (RF), Generalized Linear Model Boosting (glmBoost), Stepglm, Ridge, Elastic Net (Enet), Generalized Boosted Regression Modeling (GBM), Linear Discriminant Analysis (LDA), XGBoost, Partial Least Squares Regression for Generalized Linear Models (plsRglm), and NaiveBayes [28]. This generated 113 predictive models in total. For each algorithm, we optimized hyperparameters via 10-fold cross-validation, applying stratified sampling to split the data into training and internal validation subsets. To validate the cross-tissue robustness of the core gene signature, three independent datasets—granulosa cells (GSE137684), skeletal muscle (GSE188740), and adipose tissue (GSE84958)—were incorporated to evaluate the model’s generalizability across different PMOS-affected tissues. Model performance was then assessed by the area under the receiver operating characteristic (ROC) curve (AUC) and accuracy using the pROC package (version 1.19.0.1) [29]. Next, we applied a stacking ensemble strategy to combine the best single-model predictions. We selected the highest-confidence models and ranked their feature genes by frequency to identify candidate core genes. Finally, we visualized gene expression patterns using the ComplexHeatmap package (version 3.21.0) [30,31].

### 2.6. Model Interpretation

The internal logic of machine learning models is hard to interpret. To address this question, we turned to the SHapley Additive exPlanations (SHAP) algorithm [32]. SHAP quantified each feature’s contribution to the predictions and gave it a numerical value. Using these SHAP values, we interpretably assessed how strongly each feature influenced the model’s output.

### 2.7. Logistic Regression Analysis of Core Genes in PMOS

To evaluate the independent and combined associations of the five core genes with PMOS risk, univariate and multivariate logistic regression analyses were performed. For univariate analysis, each gene was tested individually in a separate logistic model. For multivariate analysis, all five genes were included simultaneously in a single logistic regression model to assess their independent effects after adjusting for mutual confounding. Forest plots were generated to visualize the odds ratios (OR) and 95% confidence intervals (CI) for both univariate and multivariate models.

### 2.8. Computational Analyses: Molecular Docking

We employed molecular docking to model ligand–target interactions. For AGTR1 (PDB ID: 4YAY), VCAM1 (PDB ID: 1VSC), and CYP3A5 (PDB ID: 7LAD), we retrieved their crystal structures from the Protein Data Bank (PDB). For GK5 and EGLN3, which lack experimental structures, we downloaded high-confidence AlphaFold-predicted models (average pLDDT: 93.88 and 91, respectively) from the AlphaFold Database (UniProt IDs: Q6ZS86, Q9H6Z9). All five protein structures were then docked with testosterone using CB-Dock2, a web server that performs blind protein–ligand docking, integrating cavity detection, docking simulations, and homologous template fitting [33,34]. We uploaded the 3D structure of testosterone as the ligand and sequentially docked it against each protein receptor. CB-Dock2 reports binding affinities as Vina scores, which are derived from AutoDock Vina’s scoring function (version 1.2.0). A Vina score represents predicted binding free energy in kcal/mol; more negative values indicate stronger predicted affinity. For each ligand–protein pair, we took the lowest Vina score as the primary performance metric. All docking analyses were performed in structure-based blind mode, and the pose with the lowest score was considered the most stable binding mode. We generated 3D visualizations of the resulting ligand–receptor complexes. PDB IDs and related details are summarized in Table 2.

### 2.9. Regulatory Network Analysis of Core Genes

Transcription factors (TFs) regulating the core genes were predicted using the RcisTarget package (version 1.28.1) with the cisTarget database for gene–motif rankings [35,36]. For each motif–gene set pair, the area AUC was calculated from the recovery curve, and the normalized enrichment score (NES) was derived from the AUC distribution across all motifs, with a significance threshold of NES > 3.5. Significant motifs and their associated TFs were identified, and a three-layer regulatory network (TF–Motif–Gene) was constructed. Additionally, to identify miRNAs targeting the core genes, we used three prediction databases: miRanda, miRDB, and TargetScan [37,38,39]. Only targets predicted by all three databases were considered for further analysis. In the constructed miRNA–mRNA network, the degree of each miRNA was calculated as the number of its target genes. miRNAs with a degree = 1 were removed to focus on hub miRNAs involved in synergistic regulation. Finally, the miRNA–mRNA regulatory network was visualized using Cytoscape software [25].

### 2.10. Drug Enrichment Analysis

To identify candidate drugs that may modulate the core genes and to prioritize potential therapeutic agents for repurposing, drug enrichment analysis was performed. Drug–gene interactions were retrieved from the Drug Signatures Database [40]. Drugs with *p* < 0.05 and adjusted *p* < 0.05 were considered significant.

## 3. Results

### 3.1. Identification of Testosterone Target Proteins

**Figure 2:**
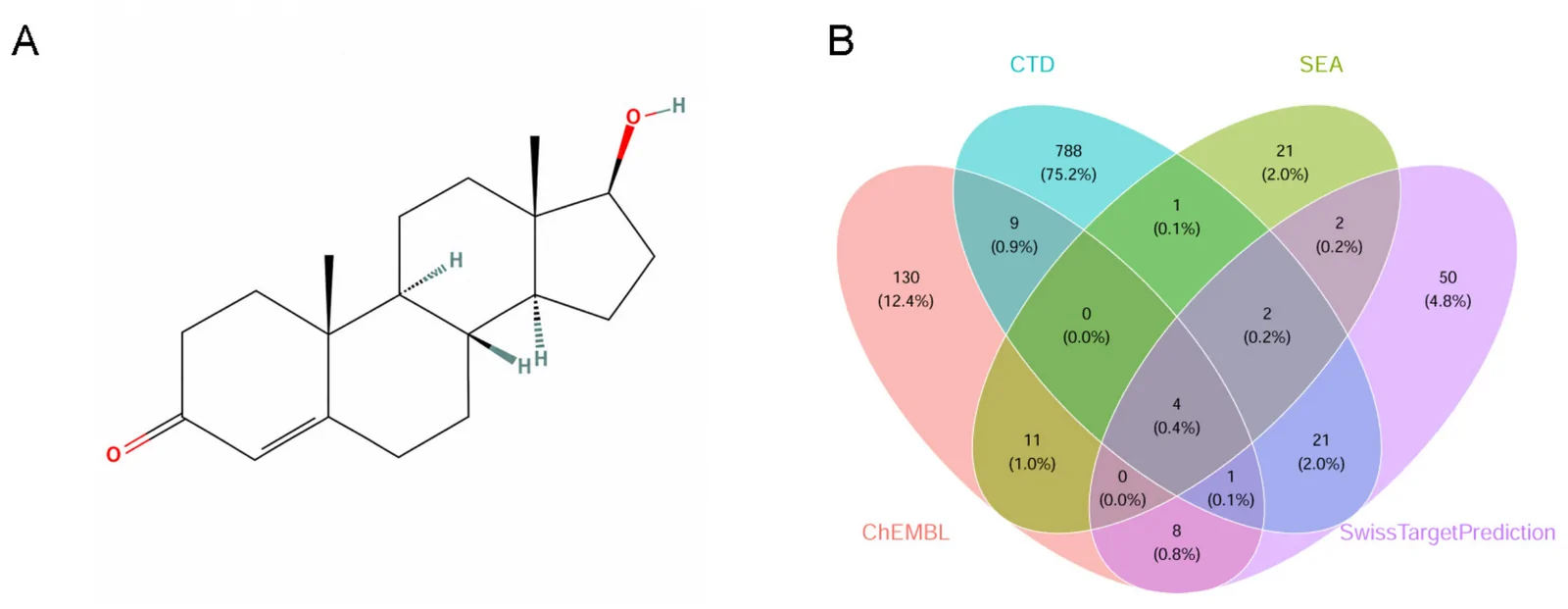
Identification of testosterone target proteins. (**A**) Chemical structure of testosterone. (**B**) Target prediction using ChEMBL, Comparative Toxicogenomics Database (CTD), Similarity ensemble approach (SEA), and SwissTargetPrediction.

The chemical structure of testosterone was obtained from the PubChem database (Figure 2A). Four complementary databases were employed to systematically forecast the potential biological targets of testosterone. After consolidating the data and eliminating duplicate records, 1048 non-redundant candidate targets were ultimately identified (Figure 2B, Supplementary Table S5).

### 3.2. Identification of PMOS-Associated Modules and Candidate Genes

**Figure 3:**
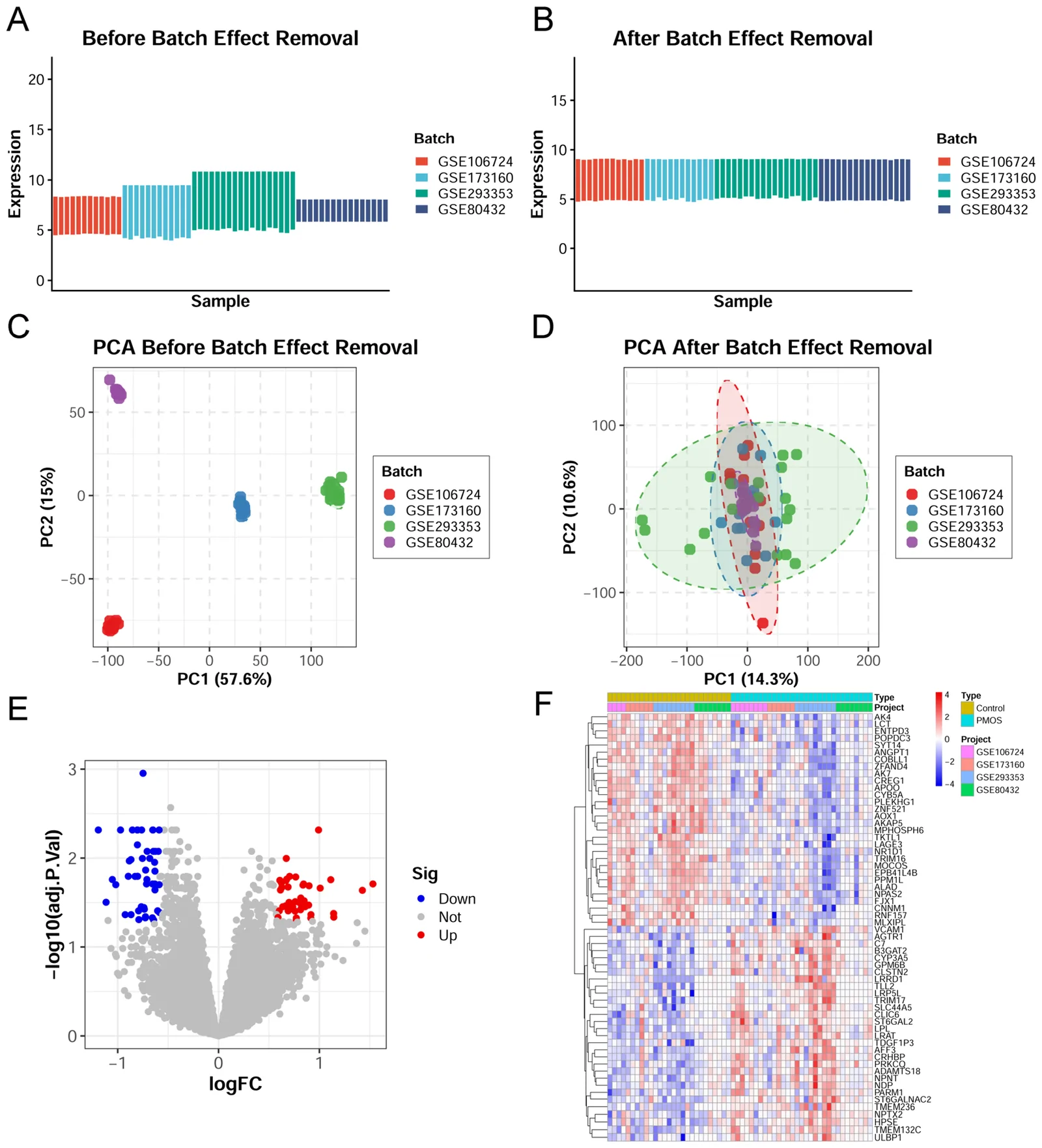
Identification of PMOS-related differentially expressed genes (DEGs). (**A**) Per-sample expression distributions prior to batch correction, with samples colored by batch origin. (**B**) Expression distributions following batch effect removal. (**C**) Principal component analysis (PCA) plot of the first two principal components before correction; ellipses indicate 95% confidence intervals (CI) per batch. (**D**) PCA plot after correction, with variance explained (%) indicated on axis labels. Dashed ellipses represent the 95% confidence intervals for each batch, illustrating the degree of overlap among batches. (**E**) Volcano plot of all genes. Red, upregulated; blue, downregulated; gray, non-significant (|logFC| > 0.585, adj.*p*.Val < 0.05). (**F**) Heatmap of top 30 upregulated and top 30 downregulated genes. Red indicates upregulation; blue indicates downregulation.

**Figure 4:**
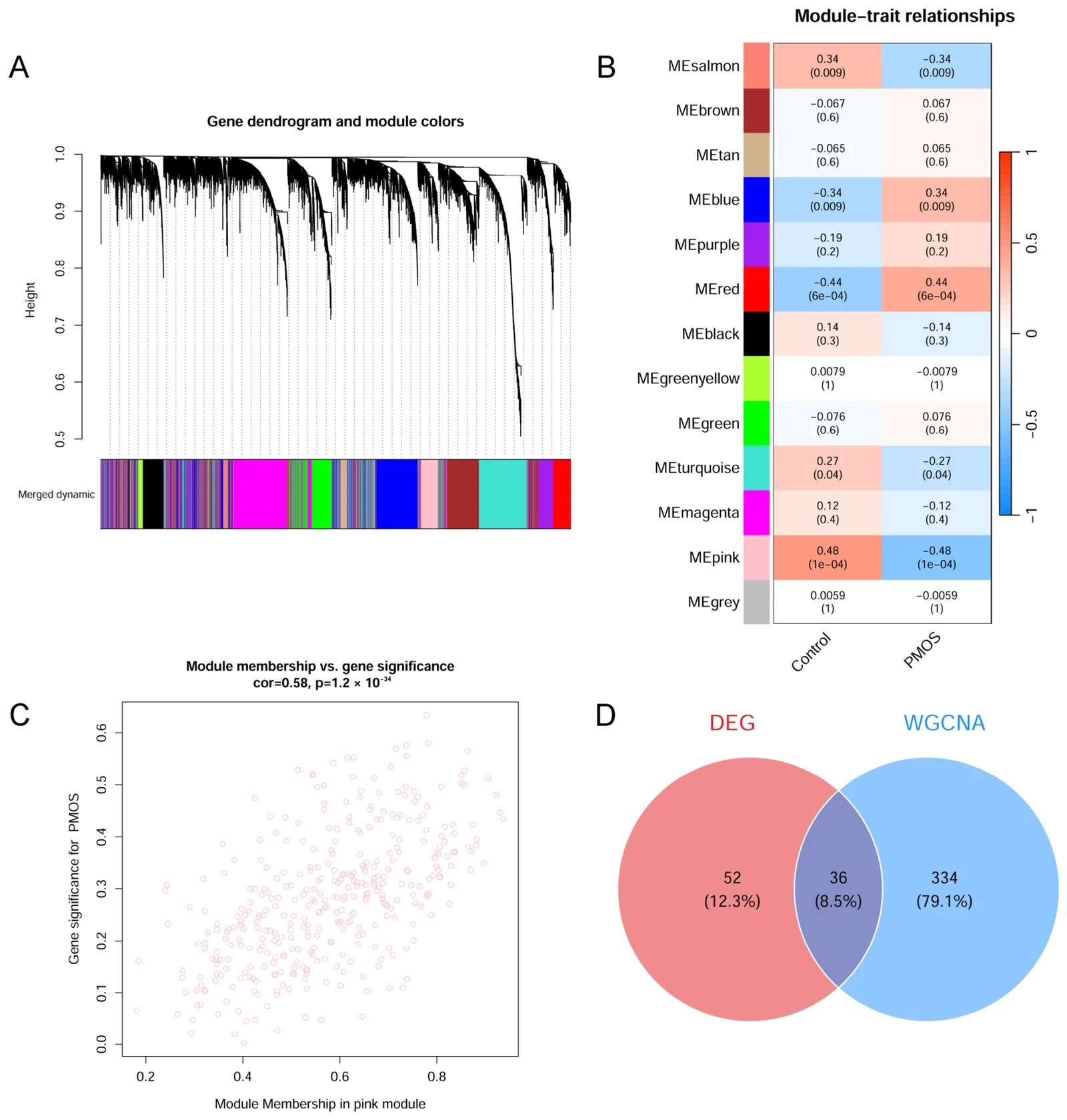
Identification of core genes for PMOS based on weighted gene co-expression network analysis (WGCNA) and DEGs. (**A**) Hierarchical clustering dendrogram of genes constructed from topological overlap matrix (TOM)-based dissimilarity. The color band below the dendrogram depicts final module assignments following module merging. (**B**) Module–trait correlation heatmap linking module eigengenes to clinical phenotypes (Control and PMOS). Blue–white–red gradient indicates correlation direction and magnitude (blue, negative; red, positive; white, zero); overlaid text displays correlation coefficients with corresponding *p*-values. (**C**) Membership significance scatter plot for the pink module, illustrating the relationship between intramodular connectivity (module membership) and trait relevance (gene significance for PMOS). (**D**) Venn diagram shows DEGs (red) and WGCNA modules (blue), with purple indicating common genes from both methods.

To address batch effects across the four datasets, we merged them and applied comprehensive normalization to the gene expression matrices (Figure 3A,B). PCA confirmed a marked improvement in data distribution after normalization; the post-normalized dataset formed more distinct clusters (Figure 3C,D). From differential expression analysis, we identified 88 genes that were significantly altered in PMOS, which we visualized using volcano plots and heatmaps (Figure 3E,F, Supplementary Table S6). Prior to network construction for WGCNA, genes with low variance (standard deviation < 0.5) were filtered out, and outlier samples were identified and removed using hierarchical clustering (cutHeight = 20,000). We first determined the optimal soft-thresholding power (β) to ensure a scale-free topology. Testing powers from 1 to 20 showed that β = 4 was the minimum value achieving the scale-free criterion (R^2^ ≥ 0.8). An unsigned network was constructed using Pearson correlation, and the TOM-based dissimilarity was calculated for hierarchical clustering. Co-expression modules were identified by dynamic tree cutting (deepSplit = 2, minModuleSize = 60), followed by merging of similar modules (cutHeight = 0.25 based on module eigengene dissimilarity). This yielded 13 distinct gene modules, each color-coded (Figure 4A). Module–trait relationship analysis revealed significant associations (*p* < 0.05) between specific modules and PMOS. Notably, the pink module showed the strongest positive correlation (Figure 4B). Within this module, MM and GS were strongly correlated with a correlation coefficient of 0.58 (*p* = 1.2 × 10^−34^), indicating a positive association between intramodular connectivity and gene significance for PMOS within this module (Figure 4C). Taking the union of pink module genes with the 88 DEGs gave 422 unique genes (Figure 4D, Supplementary Tables S7 and S8); we considered these genes to be candidate PMOS-associated genes for further analysis.

### 3.3. Identification of Testosterone-Associated Genes in PMOS

**Figure 5:**
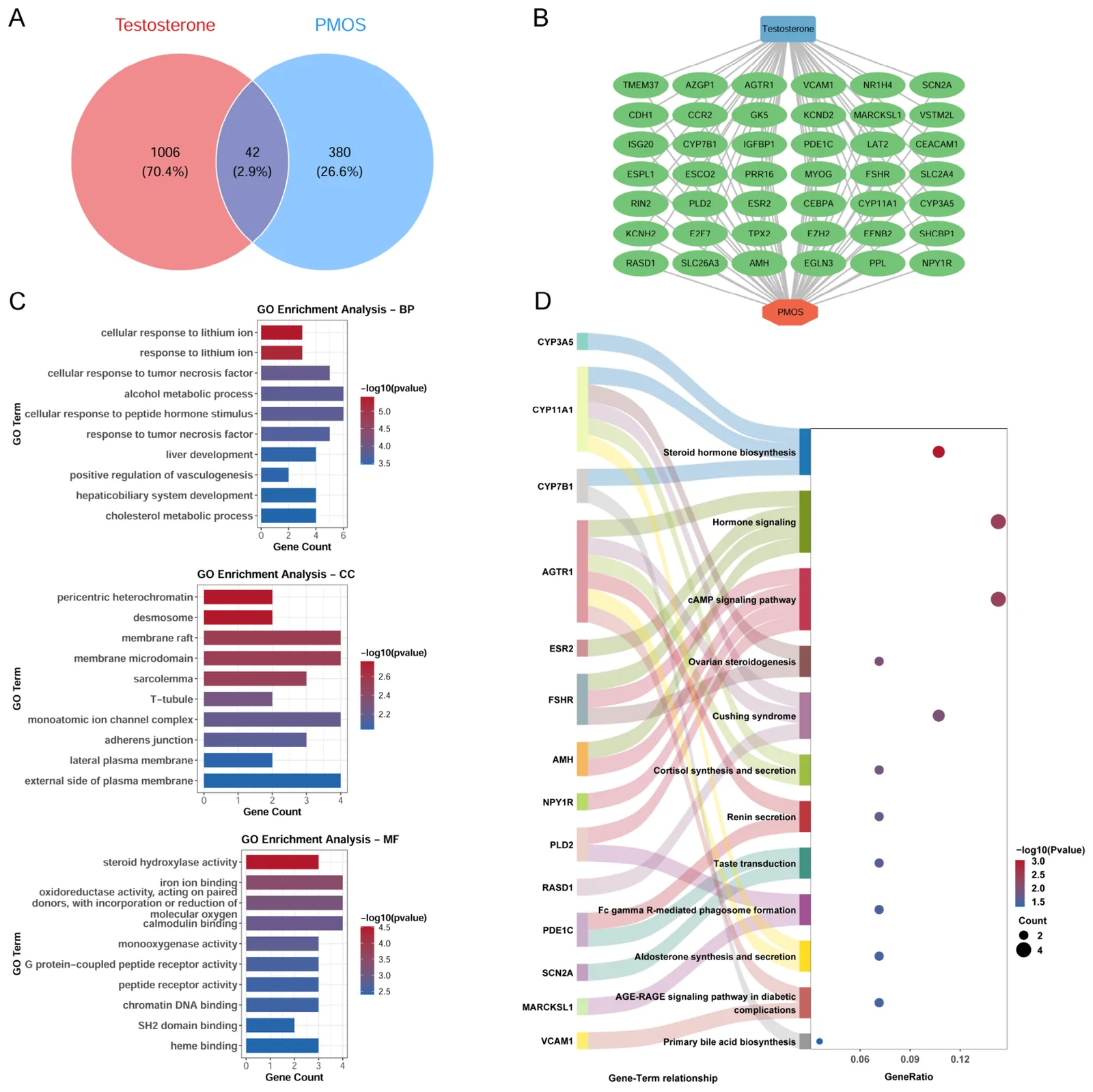
Identification of testosterone-associated genes in PMOS. (**A**) Venn diagram comparing genes linked to testosterone exposure (red) and PMOS (blue), with 42 overlapping genes (2.9%). (**B**) Testosterone–PMOS target interaction network. Blue node, testosterone; red node, PMOS; green nodes, overlapping genes linking testosterone to PMOS. (**C**) Gene Ontology (GO) enrichment analysis of overlapping genes in biological process (BP), cellular component (CC), and molecular function (MF). *X*-axis denotes gene count; color gradient represents *p*-value (darker red indicates higher significance). (**D**) Kyoto Encyclopedia of Genes and Genomes (KEGG) pathway analysis of overlapping genes. *Y*-axis shows KEGG pathway names; *x*-axis represents gene ratio. Bar length corresponds to the gene ratio associated with each pathway; color reflects enrichment significance. Scatter point position indicates gene ratio; point size is proportional to the number of genes involved in the pathway.

We intersected testosterone-associated genes with PMOS-associated genes and obtained 42 potential key targets that might be involved in the pathogenesis of testosterone-associated PMOS (Figure 5A,B). To understand their functions, we performed GO and KEGG enrichment analyses (Figure 5C,D, Supplementary Tables S9 and S10). The GO terms fell into three categories. Biological processes were significantly enriched in response to tumor necrosis factor, alcohol metabolic process, cellular response to peptide hormone stimulus, liver development, and cholesterol metabolic process. Cellular components included: membrane raft, desmosome, T-tubule, monoatomic ion channel complex, and lateral plasma membrane. Molecular functions covered: steroid hydroxylase activity, iron ion binding, calmodulin binding, G protein-coupled peptide receptor activity, SH2 domain binding, and heme binding. KEGG enrichment analysis highlighted several statistically enriched pathways. Hormonal regulation pathways included steroid hormone biosynthesis, ovarian steroidogenesis, and cortisol and aldosterone synthesis and secretion. Signaling cascades involved cAMP signaling and the AGE-RAGE signaling pathway in diabetic complications. Other relevant processes were primary bile acid biosynthesis, renin secretion, and Fc gamma R-mediated phagosome formation. Taken together, these enrichment results identified pathways related to hormonal regulation, inflammatory signaling, and metabolic processes that may warrant further investigation in the context of testosterone-associated PMOS.

### 3.4. Identification of Core Genes in Hyperandrogenism-Related PMOS

**Figure 6:**
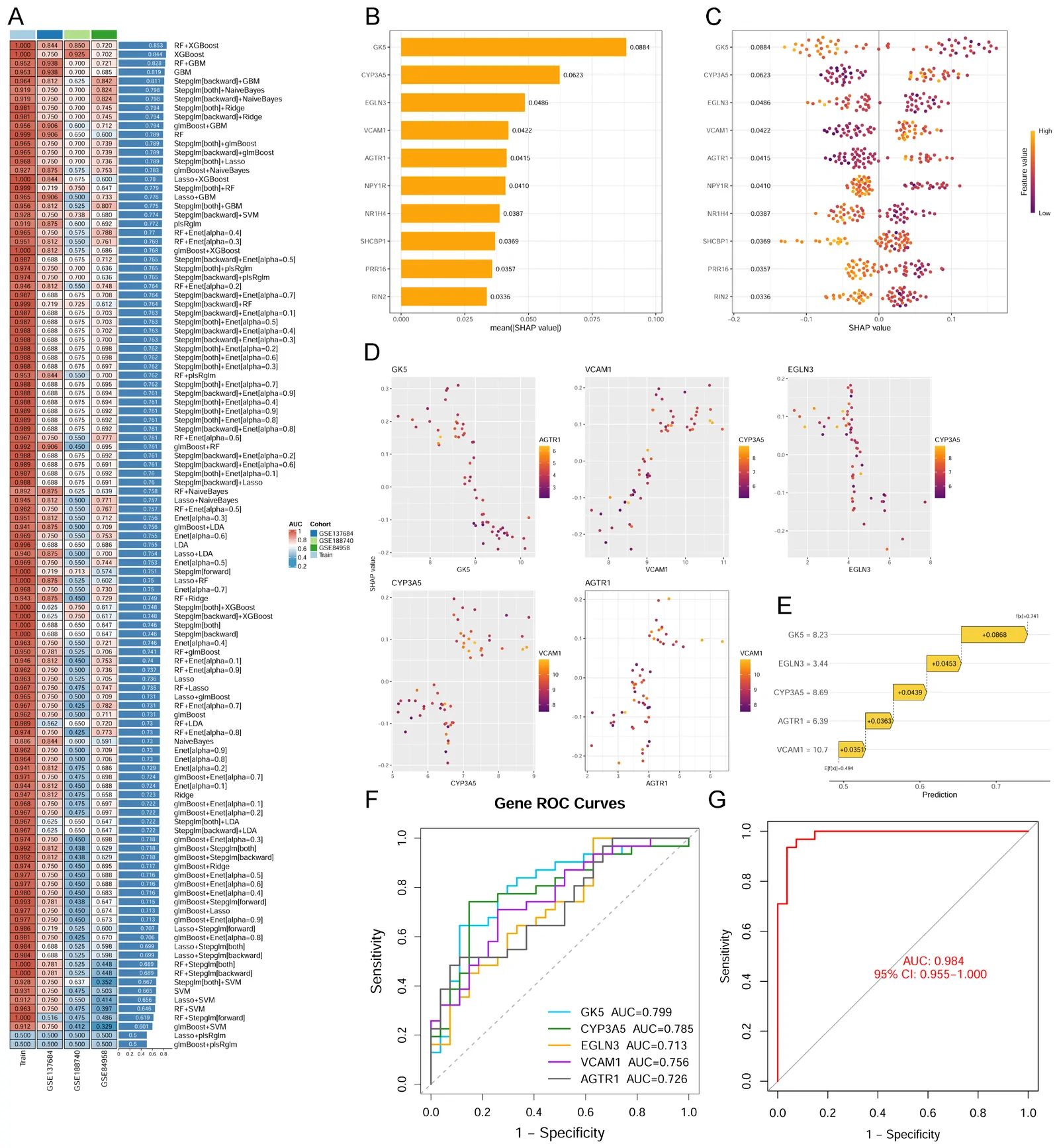
Identification of core genes in hyperandrogenism-related PMOS. (**A**) A heatmap compares model performance across cohorts. Higher area under the curve (AUC) (left column) means better prediction; colors indicate cohort origin. (**B**) A bar graph ranks genes by feature importance. Longer bars mean greater contribution to the model. GK5 and CYP3A5 stand out. (**C**) Beeswarm plot shows gene expression distributions across samples. Width reflects data density; color indicates expression level. (**D**) Scatter plot shows the distribution of SHapley Additive exPlanations (SHAP) values for the core genes. The color gradient represents the gene expression levels, indicating the interaction effects between the core genes. Positive SHAP values will cause the prediction results to tend towards a diagnosis of PMOS. (**E**) SHAP Waterfall chart shows the contribution of core genes to the prediction of individual samples. Negative SHAP = lowering effect, positive = increasing effect. (**F**,**G**) The independent and combined receiver operating characteristic (ROC) curves for five core genes (GK5, CYP3A5, EGLN3, VCAM1, AGTR1). *X*-axis: false positive rate; *Y*-axis: sensitivity. AUC values measure predictive power. The grey diagonal line represents the reference line of no discrimination (AUC = 0.5).

From the 42 candidates, we built 113 models and found that the RF + XGBoost ensemble gave the highest accuracy in training and validation (Figure 6A), prioritizing five candidate predictive features: GK5, CYP3A5, EGLN3, VCAM1, and AGTR1. SHAP analysis showed GK5 (0.0884) and CYP3A5 (0.0623) as the features with the highest SHAP importance in the selected model (Figure 6B,C). We also detected there was an inverse GK5-AGTR1 correlation and optimal CYP3A5/AGTR1 prediction at moderate expression levels (Figure 6D). GK5 (Δ = +0.0868) and EGLN3 (Δ = +0.0453) showed positive SHAP contributions to the prediction for this individual sample (Figure 6E). ROC analysis showed that the AUCs for individual genes ranged from 0.713 to 0.799, with all exceeding 0.7 (Figure 6F); In the analyzed dataset, the five-gene logistic model showed higher apparent discriminatory performance than models based on individual genes (Figure 6G). Thus, these five genes represent candidate features for diagnostic model development for testosterone-associated PMOS.

### 3.5. Logistic Regression Analysis of Core Genes in PMOS

**Figure 7:**
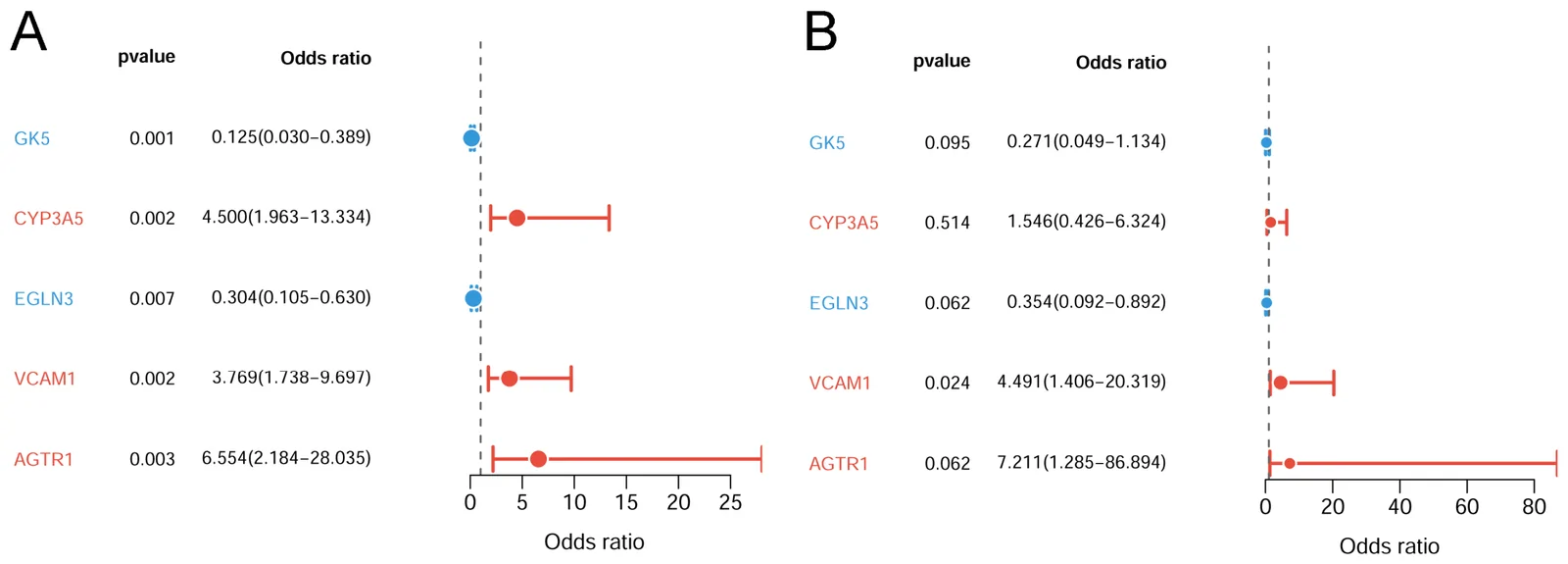
Logistic regression analyses based on core genes. (**A**) Univariate forest plot shows odds ratios (OR) with 95% CI for each gene independently associated with PMOS risk. Dots indicate point estimates; horizontal lines represent 95% CI; the vertical dashed line denotes OR = 1. *p*-values are shown for each gene. Red indicates upregulated genes, while blue indicates downregulated genes. (**B**) Multivariate forest plot displays adjusted OR with 95% CI after controlling for all genes simultaneously.

Univariate logistic regression examined each gene’s association with PMOS risk. The forest plot showed that every gene was statistically significant on its own (Figure 7A). After multivariate logistic regression with mutual adjustment of all five genes, only VCAM1 remained statistically significant in this exploratory multivariable model (Figure 7B). Although only VCAM1 remained significant, considering the possible non-linear interactions between genes and the fact that predictive factors often require multiple indicators for better reliability, the potential value of a 5-gene combined signature cannot be ruled out.

### 3.6. Molecular Docking Validation of Testosterone–Core Gene Interactions

**Figure 8:**
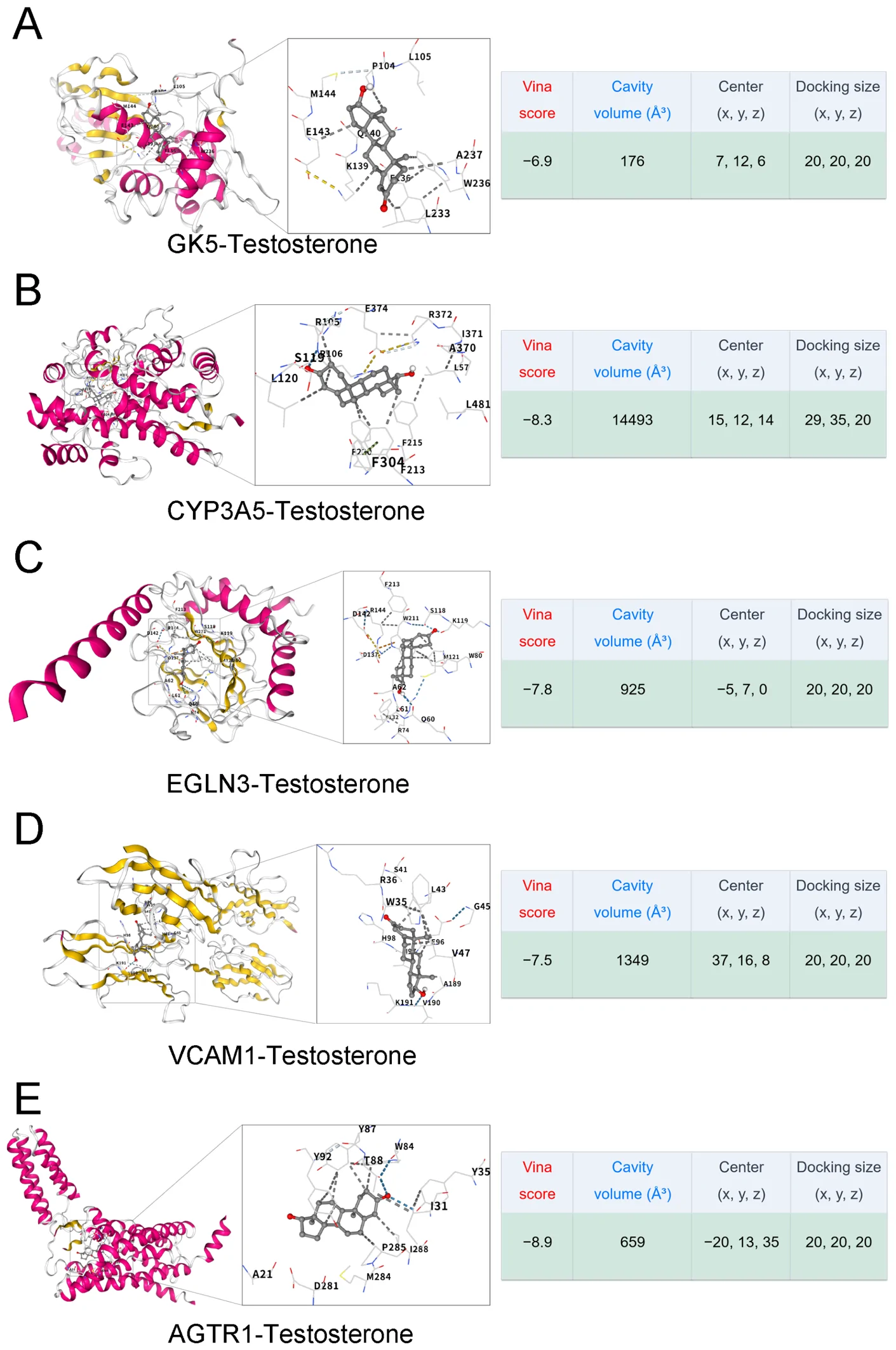
Molecular docking prediction of testosterone–core gene interactions. (**A**) Docking results of GK5 with testosterone. (**B**) Docking results of CYP3A5 with testosterone. (**C**) Docking results of EGLN3 with testosterone. (**D**) Docking results of VCAM1 with testosterone. (**E**) Docking results of AGTR1 with testosterone.

To elucidate the binding mechanisms between testosterone and its five core targets (GK5, CYP3A5, EGLN3, VCAM1, and AGTR1), we performed comprehensive molecular docking analyses using crystal structures (from PDB) and AlphaFold models. All five protein models yielded favorable docking predictions with testosterone, with binding energies consistently below −5 kcal/mol (Vina scores: −8.9 to −6.9 kcal/mol; Table 3). AGTR1 gave the strongest predicted binding, with a Vina score of −8.9 kcal/mol. Visual inspection showed that each protein model yielded a plausible docking conformation with testosterone (Figure 8). Thus, our docking results provided structural hypotheses compatible with potential physical interactions between testosterone and the PMOS-related core genes identified by machine learning.

### 3.7. Transcription Factors (TFs) Regulatory Network Analysis of Core Genes

**Figure 9:**
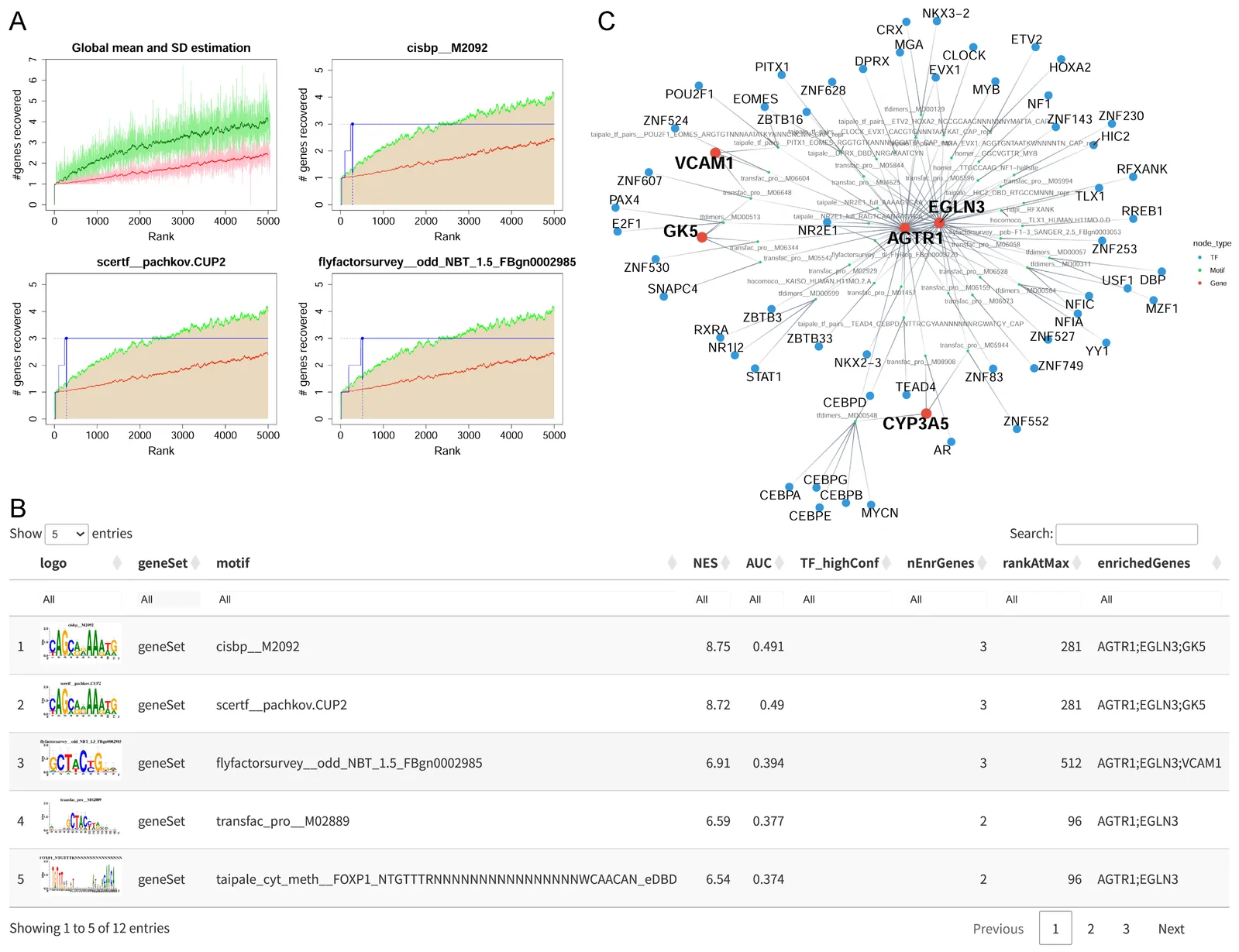
Transcription factor (TF) regulatory network analysis of core genes. (**A**) Recovery curves for the top three significantly enriched motifs. The cumulative proportion of motif-associated genes is plotted against the ranking of genes in the background set. The rapid rise in the curves indicates strong enrichment of the corresponding motifs among the core genes. The red line shows the average recovery curve across all motifs, the green line shows the mean plus standard deviation, and the blue line shows the recovery curve for the current motif. (**B**) Motif enrichment table showing the top 5 significant motifs with their normalized enrichment score (NES), AUC scores, and high-confidence TF annotations. (**C**) Three-layer regulatory network predicting the regulatory relationships between high-confidence TFs, their target motifs, and the core genes. Nodes are colored by type: blue for TFs, green for motifs, and red for genes.

To explore the transcriptional regulation of the core genes, we performed motif enrichment analysis. Multiple significantly enriched motifs were identified, which were annotated to high-confidence TFs (Figure 9A,B). A three-layer regulatory network (TF–Motif–Gene) was constructed to visualize the regulatory cascades (Figure 9C, Supplementary Table S11). The network revealed that these TFs converged on shared motifs to co-regulate the core genes. AGTR1 was associated with 39 enriched motif annotations, while EGLN3 was associated with 32 enriched motif annotations, making them the highest numbers of predicted motif associations within the regulatory network. Notably, EOMES and ZNF607 exhibited the highest regulatory breadth, each targeting three core genes. Specifically, EOMES regulated AGTR1, EGLN3, and VCAM1 through two distinct motifs, whereas ZNF607 targeted AGTR1, GK5, and VCAM1 via a single motif, suggesting their potential roles as upstream regulatory candidates. Collectively, these findings indicate that the expression of the core genes may be orchestrated by a complex transcriptional regulatory network involving multiple TFs and miRNAs, with EOMES and ZNF607 emerging as potential key regulators in this process.

### 3.8. miRNA and Drug Enrichment Analysis of Core Genes

**Figure 10:**
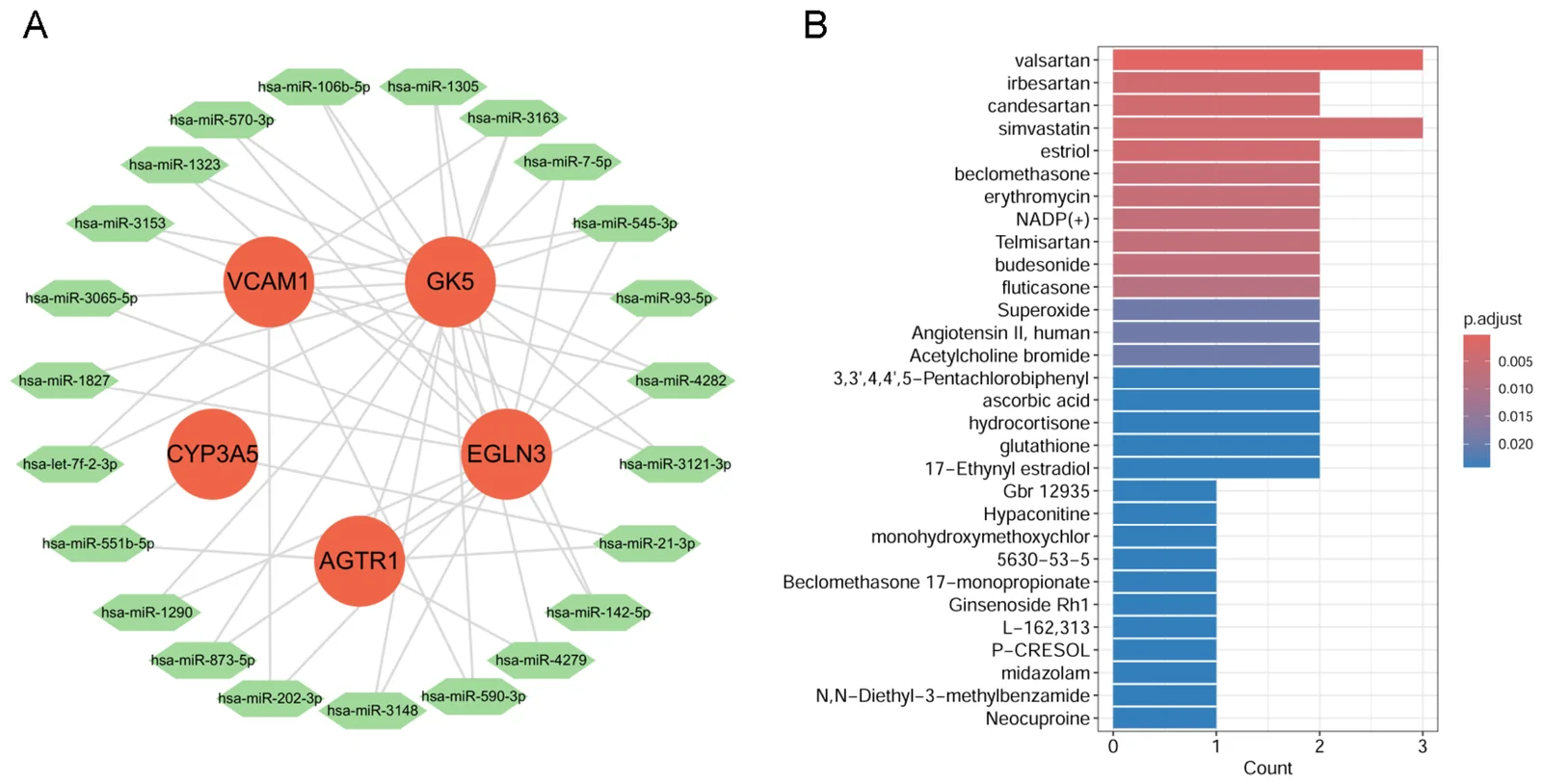
miRNA and drug enrichment analysis of core genes. (**A**) miRNA-mRNA regulatory network. Red for mRNA and green for miRNA. (**B**) Top 30 significant drugs identified by drug enrichment analysis. Bar length indicates the number of enriched genes (Count). Color represents FDR (red, more significant).

The miRNA analysis revealed that hsa-miR-3163, hsa-miR-4282, and hsa-miR-545-3p exhibited the highest regulatory capacity, each modulating three core genes. In contrast, the remaining miRNAs in the network were each connected to two target genes (Figure 10A). In addition, drug enrichment analysis of the core genes identified 184 significantly enriched drugs (Figure 10B, Supplementary Table S12). Valsartan was the most significantly enriched drug, targeting three feature genes, namely, CYP3A5, VCAM1, and AGTR1, followed by simvastatin, which also targeted these three genes. Angiotensin receptor blockers (ARBs) (irbesartan, candesartan, and telmisartan) and glucocorticoids (beclomethasone, budesonide, and fluticasone) were also significantly enriched, mainly targeting VCAM1 and AGTR1 or CYP3A5 and VCAM1.

## 4. Discussion

By integrating transcriptome data with machine learning and bioinformatics approaches, we identified potential testosterone-associated molecular targets in the pathogenesis of PMOS. Through network toxicology and bioinformatic analyses, 42 candidate genes were obtained, among which five core genes—GK5, CYP3A5, EGLN3, VCAM1, and AGTR1—exhibited the strongest predictive relevance in our models. In PMOS, GK5 and EGLN3 were downregulated, whereas CYP3A5, VCAM1, and AGTR1 were upregulated. This gene signature demonstrated high diagnostic value in machine learning models. Molecular docking additionally predicted favorable testosterone binding conformations, mediated by specific amino acid interactions. Collectively, these five genes are likely to serve as candidate mediators of testosterone-associated pathogenic mechanisms in PMOS.

PMOS is closely related to metabolic disorders such as insulin resistance, dyslipidemia, and obesity, with dyslipidemia prevalent in nearly 70% of affected women [41,42]. Patients with PMOS and hyperandrogenemia typically present with severe acne [43], which is directly related to dysfunction of the sebaceous glands. Predominantly expressed in sebaceous glands, GK5 is a skin-specific kinase that regulates sterol regulatory element-binding protein (SREBP) processing and skin lipid biosynthesis [44]. The hyperandrogenic state may indirectly affect the function or expression level of GK5 by influencing the SREBP pathway. Studies have found that lipid metabolism in granulosa cells is abnormally active in PMOS and is closely linked to insulin resistance [45,46]. Moreover, as a systemic metabolic endocrine disorder, the pathogenesis of PMOS involves interactions among multiple metabolic pathways [7], and the lipid metabolism regulated by GK5 may be closely related to the metabolic dysregulation in PMOS. Thus, the role of GK5 in PMOS is worth re-examining beyond its role as a skin-specific kinase to consider its broader involvement in the systemic metabolic regulatory network, particularly in granulosa cells, and its causal relationship with hyperandrogenemia may serve as a new entry point for unraveling the multisystem metabolic dysregulation of PMOS.

CYP3A5 catalyzes the 6β-hydroxylation of testosterone, converting it into 6β-hydroxytestosterone with significantly reduced androgenic activity, thereby participating in the clearance and inactivation of androgens in the body [47]. Moreover, the expression of this gene exhibits marked sexual dimorphism, with basal CYP3A5 expression in female hepatocytes being approximately twice that in males, which may be closely related to its special regulatory mechanisms in reproductive endocrine metabolism [48]. Notably, in endometrial tissue, CYP3A5 is highly expressed during the secretory phase of the menstrual cycle and is physiologically regulated by estrogen, suggesting that this enzyme may be widely involved in the local dynamic balance of sex hormones in the female reproductive system [49]. A previous review has pointed out that there may be a genetic link between polymorphisms of the CYP gene family and hyperandrogenemia in PMOS, but direct evidence specifically for the CYP3A5 locus remains limited [50]. On the other hand, the CYP3A5 gene is also expressed in the kidney, where it participates in blood pressure regulation by metabolizing cortisol and aldosterone, and reduced functional activity of this gene is significantly associated with elevated systolic blood pressure [51,52]. Given that PMOS patients commonly exhibit metabolic comorbidities such as hypertension [53,54], the identification of CYP3A5 as a characteristic differentially expressed gene in this study not only reflects its direct role in testosterone metabolism but also points to this gene as a potential conduit from the hyperandrogenic state of PMOS to enduring cardiometabolic risk.

EGLN3 encodes a dioxygenase that negatively regulates the activity of the HIF1 transcriptional complex by hydroxylating the HIF-1α subunit, promoting its degradation via the ubiquitination pathway, thereby maintaining cellular oxygen homeostasis [55,56]. Accumulating evidence indicates that HIF1 regulates androgen metabolism, particularly testosterone synthesis, and under hypoxia, HIF1 binds to the STAR promoter and represses its expression, thereby suppressing testosterone production [57]. Through its regulation of follicle development and release, HIF-1α also impacts female reproductive function [58]. Furthermore, activating the HIF pathway improves metabolic abnormalities in PMOS mice, such as impaired glucose tolerance, hyperglycemia, and insulin resistance [59]. Additionally, EGLN3 participates in regulating hepatic glucose and lipid metabolism and insulin sensitivity via the HIF-2α/IRS2 signaling axis, and liver-specific knockout of EGLN3 significantly improves insulin resistance [60]. Based on the above findings, EGLN3 may serve as a key hub gene linking the hyperandrogenic environment, local ovarian hypoxic stress, follicular development and ovulation, and systemic metabolic disturbances in the pathological process of PMOS. Considering its regulatory potential as an oxygen sensor in the ovarian follicular microenvironment, EGLN3 holds promise as a candidate diagnostic biomarker and a candidate therapeutic target for PMOS.

After multivariate logistic regression, only VCAM1 retained nominal significance; however, given the wide confidence intervals and model instability, validation in a larger cohort is warranted. VCAM1, a type I membrane sialoglycoprotein on cytokine-activated endothelium, mediates leukocyte–endothelial adhesion and signaling and promotes atherosclerosis [61]. Previous studies have clearly identified VCAM1 as a selective androgen target gene in theca cells, with its expression directly regulated by the AR signaling pathway [62]. In a DHEA-induced PMOS mouse model, VCAM1 has been shown to be significantly upregulated by androgens in both the granulosa cell layer and theca cell layer, and to promote local inflammatory responses by enhancing leukocyte recruitment to the ovaries, thereby forming a pro-inflammatory positive feedback loop driven by the hyperandrogenic environment [63]. Clinically, VCAM1 mRNA levels in peripheral blood mononuclear cells of PMOS patients are significantly higher than those in controls, and this difference persists after adjusting for BMI, showing a positive correlation with HOMA-IR and total cholesterol, indicating that elevated VCAM1 is directly associated with insulin resistance independent of obesity [64]. Furthermore, serum VCAM1 levels are also significantly elevated in PMOS patients and positively correlate with serum total testosterone concentrations, further confirming the upstream driving effect of hyperandrogenism on VCAM1 expression [65]. Notably, in a PMOS mouse model, conditional knockout of VCAM1 significantly suppresses cardiac inflammation and improves post-myocardial infarction cardiac injury, suggesting that VCAM1 may serve as a key molecular bridge linking the hyperandrogenic environment of PMOS to long-term cardiometabolic risk [66]. Based on the above findings, VCAM1 may participate in the chronic inflammatory state driven by hyperandrogenism in PMOS patients and, by exacerbating insulin resistance and promoting endothelial dysfunction, may further increase the risk of PMOS-related cardiovascular complications and may also serve as a novel candidate biomarker for predicting PMOS.

AGTR1 is the cell surface receptor subtype that mediates the major cardiovascular effects of angiotensin II, a key regulator of blood pressure and volume [67]. In the ovaries of DHT-induced PMOS mice, AGTR1 expression is downregulated, and its distribution is disrupted. This receptor abnormality, together with excessive activation of the ovarian renin-angiotensin system in ovarian stromal cells, may participate in the pathological processes of follicle development disorders, theca cell proliferation, and excessive androgen secretion in PMOS [68]. At the clinical level, a systematic review and meta-analysis encompassing 33 studies shows a significantly increased risk of hypertension in PMOS patients, indicating that this population faces significant long-term cardiovascular risks [69]. Importantly, in four hypertensive PMOS patients treated with an AGTR1 antagonist, serum androgen concentrations decreased significantly, and menstrual rhythm improved, suggesting that blockade of the AGTR1 signaling pathway can simultaneously alleviate both hyperandrogenemia and menstrual irregularities in PMOS [70]. Furthermore, bioinformatics analysis has clearly identified AGTR1 as a key hub protein connecting female infertility with metabolic comorbidities such as PMOS, type 2 diabetes, obesity, and cardiovascular disease [71]. Based on the above findings, AGTR1 potentially functions as an effector molecule of hyperandrogenism-related overactivation of the ovarian RAS in PMOS, and may represent a key molecular link connecting the hyperandrogenic environment, hypertension, and long-term cardiometabolic risk in PMOS.

To sum up, testosterone-associated molecular changes in PMOS may involve interconnected processes related to metabolic and endocrine regulation, cardiometabolic imbalance, and inflammatory signaling. Beyond direct targeting of the identified target proteins, our regulatory network analysis revealed that these core genes are governed by a complex transcriptional network rather than a single master regulator, involving upstream TFs and hub miRNAs—including EOMES, ZNF607, hsa-miR-3163, hsa-miR-4282, and hsa-miR-545-3p—at transcriptional and post-transcriptional levels. Furthermore, drug enrichment analysis prioritized valsartan and simvastatin as potential agents targeting CYP3A5, VCAM1, and AGTR1. To date, the use of valsartan in treating PMOS in humans has not been documented; in animal studies, only a single rat model has indicated possible benefits, including reduction of cystic follicles, amelioration of insulin resistance, improvement of lipid profiles, and lowering of testosterone [72]. Our drug enrichment analysis highlighted valsartan as a candidate for further investigation in the context of PMOS. On the other hand, simvastatin has been repurposed as a lipid-lowering agent for PMOS intervention. It reduces lipid synthesis by inhibiting HMG-CoA reductase and blocks ovarian insulin and insulin-like growth factor-1 signaling pathways. Used alone or in combination with metformin, it can significantly improve hirsutism and acne symptoms and lower total and free testosterone levels, making it a promising repurposed drug for PMOS [73,74]. Previous studies reporting potential metabolic or endocrine effects of simvastatin in PMOS provide contextual support for further investigating the drug candidates identified in our enrichment analysis. These findings suggest that, beyond their conventional use as antihypertensive and lipid-lowering drugs in clinical practice, both agents may also play a potential role as hypothetical candidates in PMOS for future experimental investigation. Furthermore, these integrated analyses provide a theoretical foundation for further mechanistic studies on hyperandrogenism-related PMOS.

This study explored hyperandrogenism-related PMOS mechanisms using public data, network toxicology, molecular docking, and regulatory networks. Several limitations should be acknowledged. First, methodological constraints exist; network toxicology and molecular docking cannot fully simulate complex biological interactions, which may affect the accuracy of the predicted genes and regulatory networks. Moreover, all pathway enrichment results indicate statistical gene clustering rather than actual pathway activation, and the identified core genes, transcription factors, miRNAs, and candidate drugs all require experimental validation to confirm their regulatory effects on testosterone-related pathological networks. Second, data availability poses a significant challenge. Our pooled granulosa cell dataset remains modest in size, reflecting the scarcity of ovarian tissue transcriptomes. Granulosa cells require invasive procurement via follicular aspiration or ovarian biopsy, and their availability is further limited by stringent ethical oversight and the heterogeneity of PMOS phenotypes. Consequently, the statistical power for detecting subtle expression changes is constrained, and the risk of model overfitting may not be completely eliminated despite our cross-validation strategies. Furthermore, feature selection was performed before cross-validation; although there was no leakage from the external validation datasets, the internal cross-validation performance may still be subject to selection-related optimism. Third, beyond these technical and data-related issues, the cross-sectional design of the study imposes inherent limitations. As a complex endocrine and metabolic disorder, PMOS may exert effects on the expression of these genes through its pathological state. Furthermore, factors such as genetic background, insulin resistance, inflammation, and obesity may have complex interactions with testosterone and gene expression. This also reflects a limitation of public databases, as the clinical information they provide is limited, making it impossible to take various confounding factors into account. Therefore, the observed associations between gene expression differences and PMOS may be bidirectional, and confounding effects from third variables may not be ruled out. In addition, PMOS encompasses four distinct clinical phenotypes, which exhibit significant heterogeneity in terms of hyperandrogenism levels and the degree of metabolic disturbances. Owing to the lack of phenotype-specific information in public databases, the present study was unable to perform stratified analyses, which may have resulted in the masking of certain phenotype-related molecular features by overall average effects. Given these limitations, future work should include both in vitro/in vivo validation of the predicted genes and longitudinal or intervention studies to disentangle causal relationships and verify actual regulatory effects. Moreover, future studies should be conducted in multicenter prospective cohorts, with phenotyping of PMOS patients and independent transcriptomic and network toxicological analyses for each phenotype, in order to uncover more precise subtype-specific pathogenic mechanisms. In addition, acquiring larger, well-characterized cohorts will be essential to confirm the robustness of our predictive signature.

## 5. Conclusions

In conclusion, our analyses identified GK5, CYP3A5, EGLN3, VCAM1, and AGTR1 as candidate molecular nodes linking testosterone-associated target predictions with PMOS-associated transcriptomic signatures. Molecular docking predicted favorable testosterone binding conformations to the identified target proteins. These findings, together with the predicted regulatory networks and candidate repurposed drugs, provide prioritized hypotheses for further mechanistic studies on hyperandrogenism-related PMOS.

## Figures and Tables

**Table 1:** GEO datasets used in the study.

Dataset	Sample Groups (Size)	Simple Type	Platform ID	Country
GSE106724	PMOS (8); Controls (4)	Granulosa cells	GPL21096	China
GSE173160	PMOS (6); Controls (6)	Granulosa cells	GPL24676	China
GSE293353	PMOS (9); Controls (9)	Granulosa cells	GPL24676	China
GSE80432	PMOS (8); Controls (8)	Granulosa cells	GPL6244	China
GSE137684	PMOS (8); Controls (4)	Granulosa cells	GPL17077	China
GSE188740	PMOS (8); Controls (5)	Skeletal muscle	GPL24676	Denmark
GSE84958	PMOS (30); Controls (23)	Adipose	GPL16791	UK

**Table 2:** PDB ID and information.

Gene	ID	Resolution A	Total Structure Weight kDa	Atom Count	Modeled Residue Count	Deposited Residue Count	Source	Average pLDDT
AGTR1	4YAY	2.9	47.68	3110	393	414	PDB	-
CYP3A5	7LAD	2.65	56.15	3528	447	481	PDB	-
VCAM1	1VSC	1.9	43.69	3359	392	392	PDB	-
EGLN3	Q9H6Z9	-	27.3	1918	239	239	AlphaFold	91
GK5	Q6ZS86	-	-	-	-	-	AlphaFold	93.88

**Table 3:** Testosterone docking prediction table. Molecular docking scores and key interacting residues between testosterone and its core protein targets. For Vina scores, more negative values predict stronger binding affinity.

Protein	Interacting Residues	Vina Score
GK5	Chain A: THR34, GLY35, PRO104, LEU105, PHE136, LYS139, GLN140, GLU143, MET144, LYS147, GLU148, CYS226, GLN227, GLU230, LEU233, GLU234, TRP236, ALA237	−6.9
CYP3A5	Chain A: LEU57, ARG105, ARG106, SER107, LEU108, GLY109, PRO110, VAL111, SER119, LEU120, PHE213, GLY214, PHE215, PHE220, LEU240, PHE241, ILE301, PHE304, ALA305, THR309, ALA370, ILE371, ARG372, LEU373, GLU374, GLN479, GLY480, LEU481	−8.3
EGLN3	Chain A: ALA55, ARG57, ASP58, GLY59, GLN60, LEU61, ALA62, LEU73, ARG74, ILE78, THR79, TRP80, ARG117, SER118, LYS119, MET121, TYR132, VAL133, ARG134, HIS135, VAL136, ASP137, PRO139, ASP142, ARG144, TRP211, PHE213, ARG218	−7.8
VCAM1	Chain A: PHE32, PHE33, SER34, TRP35, ARG36, THR37, ASP40, SER41, PRO42, LEU43, GLY45, LYS46, VAL47, ASN49 Chain B: ASP94, PRO95, GLU96, ILE97, HIS98, LEU99, GLN188, ALA189, VAL190, LYS191	−7.5
AGTR1	Chain A: LEU13, ASN14, SER15, SER16, CYS18, PRO19, LYS20, ALA21, GLY22, ARG23, ILE31, TYR35, PHE77, LEU81, TRP84, TYR87, THR88, TYR92, SER105, VAL108, ARG167, ILE172, VAL179, CYS180, ALA181, PHE182, HIS256, THR260, ASP263, ASP281, MET284, PRO285, ILE286, ILE288, TYR292	−8.9

## Data Availability

Data are contained within the article and Supplementary Materials; public sources are available as described in the article.

## Supplementary Materials

The following supporting information can be downloaded at: https://www.mdpi.com/article/10.3390/metabo16090663/s1, Supplementary Table S1. Testosterone target retrieval result files from the ChEMBL database. Supplementary Table S2. Testosterone target retrieval result files from the SwissTargetPrediction database. Supplementary Table S3. Testosterone target retrieval result files from the SEA database. Supplementary Table S4. Testosterone target retrieval result files from the CTD database. Supplementary Table S5. Final testosterone target library integrating target data from the four databases. Supplementary Table S6. Differentially expressed genes of PMOS from the GEO database. Supplementary Table S7. List of genes in the pink module identified by WGCNA. Supplementary Table S8. Intersection genes between DEG and WGCNA. Supplementary Table S9. Significant terms from GO enrichment analysis. Supplementary Table S10. Significant pathways from KEGG pathway enrichment analysis. Supplementary Table S11. Regulatory interaction list of transcription factors, motifs, and target genes in the three-layer network. Supplementary Table S12. Significant drugs identified by drug enrichment analysis of key genes.

## Abbreviations

The following abbreviations are used in this manuscript:

PMOS	Polyendocrine metabolic ovarian syndrome
PCOS	Polycystic ovary syndrome
GEO	Gene Expression Omnibus
PCA	Principal component analysis
WGCNA	Weighted gene co-expression network analysis
TOM	Topological overlap matrix
MM	Module membership
GS	Gene significance
DEGs	Differentially expressed genes
GO	Gene Ontology
KEGG	Kyoto Encyclopedia of Genes and Genomes
AUC	Area under the ROC curve
ROC	Receiver operating characteristic
SHAP	SHapley Additive exPlanations
OR	Odds ratio
CI	Confidence interval
PDB	Protein Data Bank
TF	Transcription factor
NES	Normalized enrichment score
SREBP	Sterol regulatory element-binding protein
